# Experimental Investigation on Red Mud from the Bayer Process for Cemented Paste Backfill

**DOI:** 10.3390/ijerph191911926

**Published:** 2022-09-21

**Authors:** Jiwei Bian, Shuai Li, Qinli Zhang

**Affiliations:** School of Resources and Safety Engineering, Central South University, Yuelu District, Changsha 410083, China

**Keywords:** red mud, CPB, water resistance, leaching

## Abstract

Red mud is a by-product of alumina production, and its disposal can have severe environmental consequences. This study experimentally investigates the feasibility of using red mud from the Bayer process for cemented paste backfill (CPB). Different binders and activators were used to improve the mechanical properties, water resistance, and environmental behaviors of red mud-based CPB. In addition, water immersion tests were introduced, for the first time, to evaluate the water resistance of CPB. Furthermore, the environmental behaviors of red mud-based CPB were investigated by conducting leaching experiments. The results showed that the red-mud specimens had an unconfined compressive strength (UCS) of less than 0.2 MPa and disintegrated after being immersed in water. Different binders significantly improved the mechanical properties of red mud-based CPB. In addition, the specimens with different binders showed excellent water resistance, and the softening coefficient of CPB with different binders could exceed 0.7 after being cured for 28 days. The binders exhibited a substantial inhibitory effect on the leaching of hazardous substances in red mud under the solidification and stabilization effects. The leaching concentration of hexavalent chromium, selenium, fluoride, arsenic, lead, and vanadium was reduced by more than 70%. Therefore, this study provides an effective method for the environmental-friendly and large-scale utilization of red mud from the Bayer process.

## 1. Introduction

**Figure 1:**
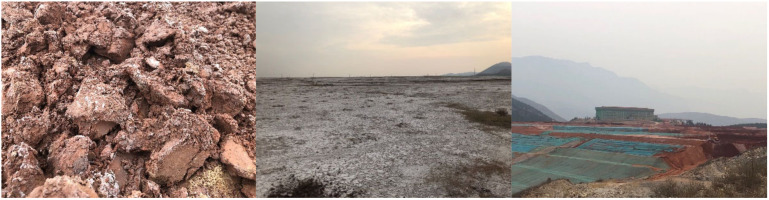
Open-air storage yard of red mud.

Red mud is a type of red, silty, and strong alkaline solid waste generated in the process of alumina extraction from bauxite [1,2]. Generally, 1.0–1.8 tons of red mud is produced per ton of alumina [3], depending on the quality of bauxite. More than 70 million tons of red mud is produced annually in China [4,5], and the cumulative amount of red mud has exceeded 352 million tons. The growth of alumina production in China and the gradual reduction of bauxite grade indicate that the annual production of red mud will continue to increase. Traditionally, fresh red mud would be directly discharged and stored in the open-air storage yard (Figure 1) without any further treatment, which not only occupies a large area of land but also poses safety hazards to the surrounding environment [6,7]. The alkaline liquid leached during long-term storage penetrates the ground, polluting the water sources and causing soil alkalization [8,9]. In addition, the exposure of red mud in the air forms dust, which severely pollutes the atmosphere. With the implementation of environmental protection policies and the emphasis on sustainable development, the disposal of red mud has become a critical bottleneck, restricting the development of the alumina industry and placing a heavy burden on socio-economic development and environmental protection. Therefore, an efficient disposal and comprehensive utilization of red mud must be prioritized.

Following alumina production processes, red mud can be categorized into red mud from the Bayer process, red mud from the sintering process, and red mud from the combined process [10,11]. There are some differences in the physical properties and chemical compositions of red mud produced from different bauxite sources and alumina production processes [12]. The main chemical compositions of red mud are similar, mainly including SiO_2_, Al_2_O_3_, CaO, Fe_2_O_3_, TiO_2_, Na_2_O, K_2_O, and MgO, etc., but the content of various compositions is different due to the alumina production processes. The active ingredients of red mud from the sintering process and the combined processes would show potential hydration properties. However, the contents of soluble alkali (sodium hydroxide, sodium aluminate, and sodium carbonate) and insoluble alkali (sodium aluminosilicate hydrate) in red mud from the Bayer process are high. In addition, there are almost no active ingredients. Thus, this type of red mud shows low stability and permeability.

Extensive research efforts have been made toward investigating comprehensive utilization technologies of red mud, and some methods and solutions have been proposed from the perspectives of building materials, resource recycling, environmental protection, and biological treatment. Considering its potential hydration properties, red mud from the sintering process and the combined process can be used for producing cement [13,14], building bricks [15], glass, alkali-activated materials [16], and special ceramics [17], as well as additives or excipients for asphalt materials, roadbed materials [18,19], thermal insulation materials, and other building materials. In addition, some valuable metal elements, especially rare ones, can be extracted from red mud by the leaching process or a roasting method [20,21,22,23]. Red mud with a large specific surface area shows excellent adsorption capacity for metal ions and radioactive elements, which can be used as an adsorbent for environmental restoration, such as waste gas treatment, wastewater treatment, and soil remediation [24,25,26]. In addition, red mud is used to produce coal-burning desulfurizers, polymer water-purifying agents, and siliceous calcium agricultural fertilizers. Therefore, most researchers have focused on red mud from the sintering process and the combined processes, while the research on red mud from the Bayer process is still in its infancy. Furthermore, these researches on red mud are mostly only technically feasible. However, the complexity of the process technology and the relatively low value-added products make it challenging to achieve excellent economic benefits. There are a large number of alkaline compounds (Na_2_O and K_2_O) that are difficult to eliminate, as well as fluorine, aluminum, and many other impurities, which pose an environmental risk in the preparation of building or functional materials. Thus, the contradiction between technology and the economy, as well as safety and environmental protection issues, means the comprehensive utilization of red mud, still in the experiment and research stage, is limited in its industrial application and large-scale popularity. Consequently, the comprehensive utilization rate of red mud is less than 4% in China, which, compared to its enormous emissions, still cannot reduce its heavy burden on society and the environment. Therefore, it is exceptionally urgent to minimize the hazards of red mud and achieve its multichannel and large-scale utilization.

Cemented paste backfill (CPB), which is a mixture of solid waste, binder, and water, is transported from the surface to underground voids in the form of backfilling slurry by gravity or pumping and then consolidated into a block with mechanical strength [27,28,29]. It not only improves the safety of the underground mining environment but also prevents surface subsidence and can fully utilize the solid waste disposed on the surface [30,31]. Owing to its numerous economic and environmental advantages, CPB is considered an effective and practical tailings management strategy, and thus has been widely adopted in mines all over the world during the past few decades. Generally, the utilization rate of tailings for CPB is 50–60%, and even 100% in some mines. Technically, CPB technology can consume considerable red mud. The hardened CPB, an artificial structure in underground mining, acts as a temporary or permanent pillar to support the surrounding rock, and thus must meet the dynamic and static loading requirements to ensure the safety of the mining environment [32,33]. Mechanical properties are the most crucial design criterion for CPB, depending on the mining conditions and function of CPB. The unconfined compressive strength (UCS) tests performed on the laboratory specimens cured in conventional plastic molds at atmospheric pressure and temperature are the most common and economical methods for assessing the mechanical properties of CPB. Belem and Benzaazoua [34] summarized numerous previous studies and found that the UCS of CPB generally varies from 0.2 to 4 MPa. When red mud is used as a backfilling aggregate, the strength development of CPB should be studied to satisfy the strength requirements of mines. Meanwhile, environment behavior is another fundamental performance characteristic of CPB. There are a lot of residual alkalis, heavy metal ions, and fluoride ions in red mud that would migrate with water. Previous research [35,36,37] has shown that CPB can play a functional role in stabilizing/solidifying the impurities. Nevertheless, the high contents of impurities in red mud make it essential to evaluate the environmental behavior of red mud-based CPB.

In an environment-friendly, economically reasonable, and technically feasible manner, CPB provides a reference for the large-scale disposal and utilization of red mud. Zhu et al. [38] used red mud from the sintering process as a partial replacement of binders in CPB practices. Owing to the unique physicochemical properties of red mud from the Bayer process to date, no study has been conducted on CPB formed using such a type of red mud. Thus, a sufficient understanding of the properties and performances of this type of CPB is required before its practice and popularization. This study aimed to experimentally explore the feasibility of using red mud from the Bayer process as a backfilling aggregate. Different binders are used to improve the properties of this type of red mud, which are expected to not only make its mechanical properties meet the relevant technical requirements but also prevent secondary pollution in the environment during the application process.

## 2. Materials and Methods

### 2.1. Materials

Generally, CPB is a homogeneous and engineered mixture produced by tailings, binder, water, which is either fresh or mine-processed, and additives.

(1) Tailings

**Figure 2:**
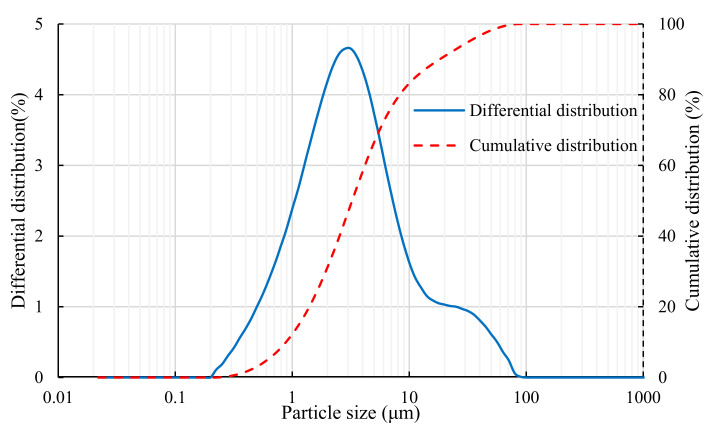
Particle size distribution of red mud from the Bayer process.

**Figure 3:**
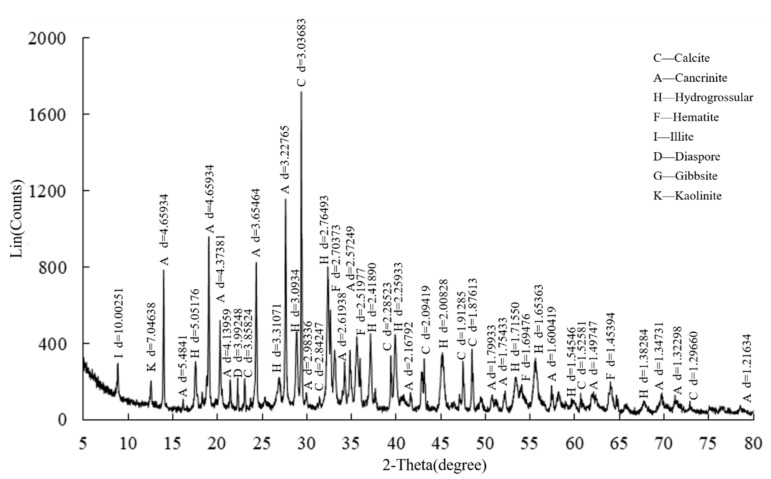
XRD results of red mud from the Bayer process.

A typical red mud from the Bayer process, utilized in this study, was obtained from an aluminum industry Co. Ltd. in Jiaozuo City, Henan Province, China. The physical properties and chemical compositions of tailings are well known to have a substantial influence on the mechanical properties of CPB [39]. In this study, the dried red mud was thoroughly pulverized by wood milling, and then, the powder specimens were subjected to a particle size analysis by using a laser diffraction particle size analyzer (Mastersizer 2000, Malvern, United Kingdom). The particle size distribution of red mud from the Bayer process is presented in Figure 2, and its primary physical properties are listed in Table 1. Red mud has a fine particle size distribution, with particles less than 5 μm accounting for 66% and particles larger than 38 μm accounting for only 4%. Thus, red mud can be classified as ultrafine tailings based on the unified soil classification system. In addition, red mud has a large specific surface area of 2940 m^2^/kg, which is much higher than that of the ordinary binder. In addition, the high content of fine particles in red mud leads to a low permeability coefficient (3.35 × 10^−7^ cm/s). These properties are expected to cause some difficulties in the dehydration and strength development of red mud-based CPB. In addition, the nonuniform coefficient is less than 5.0 and the curvature coefficient is less than 1.0, which indicates that the gradation of red mud is poor. The water content of fresh red mud exceeds 40%, and the pH value of the squeezed filtrate reaches 12.1. All of these would hinder the strength development of CPB. It is well-known that the chemical compositions of tailings obtained from different mines affect the hydration processes and products, causing uncertainties in CPB. The chemical compositions of red mud from the Bayer process, listed in Figure 3, were determined by X-ray diffraction (XRD). The mineral compositions, including calcite, cancrinite, hydrogrossular, hematite, gibbsite, mica, and chlorite, are complex and do not have potential hydration properties. Moreover, fluoride and heavy metal ions, such as Pb, Cr, and As, were detected, which may impact the groundwater environment. In terms of physical properties and chemical compositions, the engineering characteristics of red mud from the Bayer process are poor, and thus it is not an ideal backfilling aggregate.

(2) Binder

A binder refers to a powder material that can firmly bond other materials into a block with mechanical strength through its hydration reactions. Cement is a hydraulic inorganic cementitious material, and its hydration reaction plays a decisive role in the development of CPB structure. Portland cement, the most common binder in CPB practices, was used in the study; its main chemical compositions are listed in Table 2. The specific gravity of this cement is 2.86 g/cm^3^, and its specific surface area is 364 m^2^/kg.

Slag is an industrial solid waste produced in the pyrometallurgical process. Its main chemical components include CaO, Fe_2_O_3_, Al_2_O_3_, MgO, and other alkaline oxides [40,41]. Slag shows potential hydration properties and can react with calcium hydroxide or another hydroxide in the presence of moisture and activators. It can partly or fully substitute cement in CPB practices, improving the gradation of backfilling materials and enhancing the mechanical properties at an advanced age. Slag V500 (S95), a granulated blast furnace slag, was used in this study. The chemical properties of the slag are listed in Table 2; it has a density of 2.83 g/cm^3^ and a specific surface area of 396 m^2^/kg.

(3) Activator

The volcanic ash materials and potential hydration materials hardly hydrate with water in the absence of an activator. Lime was used as an activator in the study. Lime is a gas-hard inorganic cementitious material whose main composition is calcium oxide, and it is strongly alkaline.

### 2.2. Methods

#### 2.2.1. Experimental Programs

(1) Self-consolidation characteristics of red mud

Red mud from the Bayer process undergoes an apparent phenomenon in which it self-consolidates into blocks. Therefore, an experiment was conducted to observe the self-consolidation characteristics of this type of red mud. The specimens were only made of red mud, without any binders and additives.

(2) Cementitious characteristics of the binders

By analyzing the physical and mechanical properties of red mud from the Bayer process, it was found that its engineering characteristics are poor, and thus it cannot be directly applied to engineering practices. Therefore, cement and slag were added as modified materials to improve its mechanical properties and water resistance through a series of physical and chemical reactions.

(3) Cementitious characteristics of binders with activators

Lime is used to improve the bonding effect of binders or activate their potential hydration properties. The amount of lime added in this study was 6% of that of the binders and lime.

#### 2.2.2. Specimen Preparation

According to the above-discussed experimental programs, more than 200 specimens were prepared, all of which had a constant tailing–binder ratio of 6.0 and a solid mass concentration of 60%. A specific amount of binder, red mud from the Bayer process, and tap water were mixed in a mixing tank for seven minutes until a homogeneous slurry was obtained. Then, the slurry was poured into standard plastic molds with a diameter of 5 cm and a height of 10 cm. The air trapped in the specimens was removed by manual vibration, and the specimens were cured for 3, 7, and 28 days at room temperature (20 ± 2 °C).

#### 2.2.3. Experimental Methods

(1) UCS tests

The specimens cured for 3, 7, and 28 days were subjected to UCS tests by using a WDW-2000 rigid hydraulic pressure servo-machine following the “Standard test method for compressive strength of cylindrical concrete specimens” (ASTM C39). The loading was applied at a displacement rate of 0.5 mm/min, and the strain and corresponding loading were monitored and recorded. All tests were repeated at least three times to ensure the repeatability of the results, and the average was considered as the UCS value of the specimens.

(2) Water immersion tests

CPB would inevitably suffer from immersion and leaching of groundwater in the underground voids. Its microstructure and properties will be affected by the environment. Therefore, CPB should exhibit excellent durability during its service life. In this study, water immersion tests were conducted to evaluate the performance of CPB. Water resistance refers to the ability of CPB to resist water damage [42]. The water resistance of red mud-based CPB was analyzed by comparing the UCS under unfavorable conditions (water immersion) and standard conditions (non-immersion). Usually, the softening coefficient is used to indicate the water resistance of CPB and can be calculated with Equation (1). The mechanical properties of CPB with strong water resistance do not easily deteriorate.

(1) $$k=\frac{f_{1}}{f_{0}}$$

where k is the softening coefficient; *f*_1_ is the UCS of red mud-based CPB immersed in water, MPa; and *f*_0_ is the UCS of that cured under standard conditions, MPa.

According to the “Technical standard for application of soil stabilizer” (CJJ/T286-2018), the specimens were immersed in a water bath for 24 h before conducting the UCS tests. The water surface was at least 2.5 cm higher than the top surface of the specimens, and the immersing temperature was 20 °C.

(3) Leaching experiments

Besides high alkalinity, red mud from the Bayer process also contains a lot of heavy metal ions, such as Pb, Cr, and As, as well as fluoride. The soluble impurities in red mud would dissolve in water during long-term immersion, which may pose substantial potential hazards to the groundwater environment. To prevent secondary pollution, the environmental behaviors of red mud-based CPB should be evaluated before its application and popularization.

Leaching experiments are used to simulate the leaching process of harmful impurities under the influence of groundwater after CPB is filled into underground voids. Following the “Identification standard for hazardous wastes—identification for extraction toxicity” (GB5085.3-2007), the leaching toxicity of the hazardous impurities in the specimens was determined. The leaching experiments were conducted on fresh red mud from the yard and the debris destroyed in the UCS tests.

(4) Microstructural analysis

The mechanical properties of CPB are dependent on its microstructure [43,44,45]. To visually observe the microscopic morphology of hydration products, the microstructure of red mud-based CPB was analyzed by scanning electron microscope (JSM-IT500). The specimens destroyed in the UCS tests were immersed in absolute ethanol for 24 h to terminate the hydration reactions and then analyzed after the solutions were removed.

## 3. Results and Discussion

### 3.1. Strength Development of Red Mud-Based CPB under Standard Conditions

#### 3.1.1. Strength Development of Specimens with Red Mud

**Figure 4:**
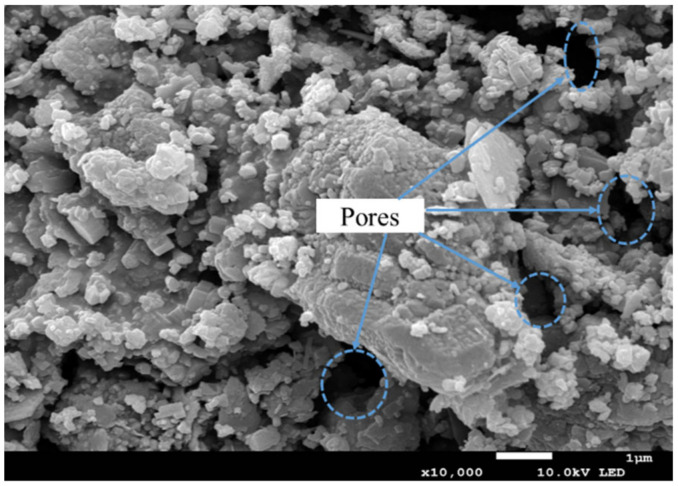
SEM micrograph of CPB with red mud.

The specimens prepared only with red mud from the Bayer process had not consolidated in 3 days; they began to consolidate on the fifth day. However, the specimens showed low UCS values (only 0.12 MPa in 7 days and 0.15 MPa in 28 days). The consolidation of red mud specimens is primarily attributed to the interaction between red mud particles. The interaction depends on the combined effect of attractive and repulsive forces, including electrostatic forces, van der Waal’s forces, capillary forces, and cementation [46,47,48]. (1) Red mud particles are negatively charged and can adsorb cations from aqueous solutions. However, the charges are not evenly distributed on the surface of these particles. When particles with different charges are close to each other, they would be attracted by electrostatic forces. In addition, there is an electric double layer on the surface of red mud particles. When particles are sufficiently close, these double layers of adjacent particles will overlap, forming a commonly bound water film. Thus, bound water around the particles can play a key role in bonding adjacent particles. The bonding strength is dependent on the thickness of the bound water. (2) Van der Waal’s force is a universal interaction force between particles. As the distance between the particles increases, the forces gradually weaken. The particles collide with each other continuously by Brownian motion and gradually adhere to each other by electrostatic and Van der Waal’s forces to form a cohesive structure. (3) With the consumption and evaporation of free water, a large number of interconnected capillary pores are generated inside the specimens. The free water left at the particle contact forms a concave meniscus under the surface tension, thereby generating capillary pressure [49,50]. In turn, the particles are subjected to reaction forces, i.e., effective compressive stress, which produces a frictional force between particles [51,52]. The frictional forces appear to be cohesive, encouraging the particles to agglomerate tightly. When the specimens are saturated or dried, the capillary forces would disappear. (4) The cementitious substances bound the dispersed particles, which is considered to be a chemical bond. Red mud contains several clay minerals (kaolin and illite) and carbonate minerals (calcite), which provide a bonding effect. Based on the cohesive structure, the dispersed particles are connected to form a hardened structure by cementation. Therefore, the consolidation of the specimens is a process in which these interactions agglomerate the particles into a block. As shown in Figure 4, the specimen structure was relatively loose and there were small pores in the SEM micrograph. It is also hard to find C-H, C-S-H or AFt in Figure 4. That is to say, the particles of different shapes and sizes are consolidated into a block under the combined effect of various forces.

#### 3.1.2. Strength Development of Red Mud-Based CPB with the Binder

**Figure 5:**
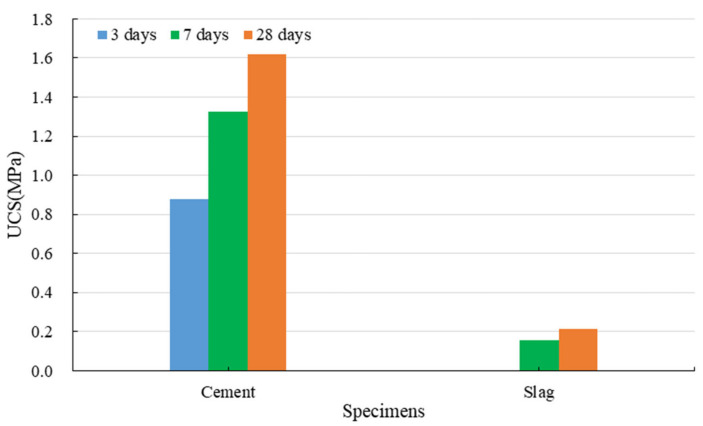
Strength development of red mud-based CPB with different binders.

**Figure 6:**
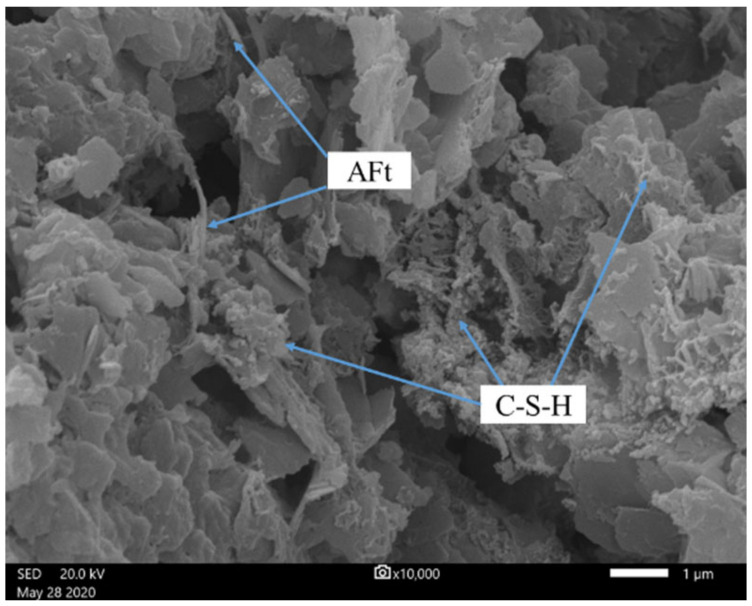
SEM micrograph of CPB with cement.

Figure 5 indicates the strength development of red mud-based CPB with different binders under standard conditions. The UCS of CPB with cement is significantly increased and can reach 1.4 MPa in 3 days. This is mainly due to the hydration reactions of cement. Compared to red mud specimens, the cementation produced by cement is more significant. Portland cement is a multimineral aggregate composed mainly of four clinkers. It can produce extremely complicated reactions during the hydration processes and generate considerable hydration products (C-S-H gel, CH, and AFt), filling the space previously occupied by capillary water [53,54]. This increases the volume of the solid phase in the system and shortens the distance between solid particles. The hydration products overlap and cross each other, connecting the previously dispersed red mud particles to form a three-dimensional network structure gradually. As the hydration reactions progress, the amount of hydration products is continuously increased, and the crystal size continues to grow. The precipitation of hydration products can compact and refine the pore structures, significantly reducing the porosity of CPB [55,56]. These make the structures denser and more stable. In addition, the alkaline system of red mud is beneficial for stabilizing the C-S-H gel. Relying on the hydration reactions of cement, the backfilling slurry gradually develops into a hardened structure with the ability to resist external stress. This is demonstrated by the results of SEM on the specimens with cement cured for 28 days. As we can see in Figure 6, hydration products can be found everywhere. A large number of fibrous/spherical C-S-H cover on the particle surface. In addition, there is a small amount of rod/needle-like AFt.

**Figure 7:**
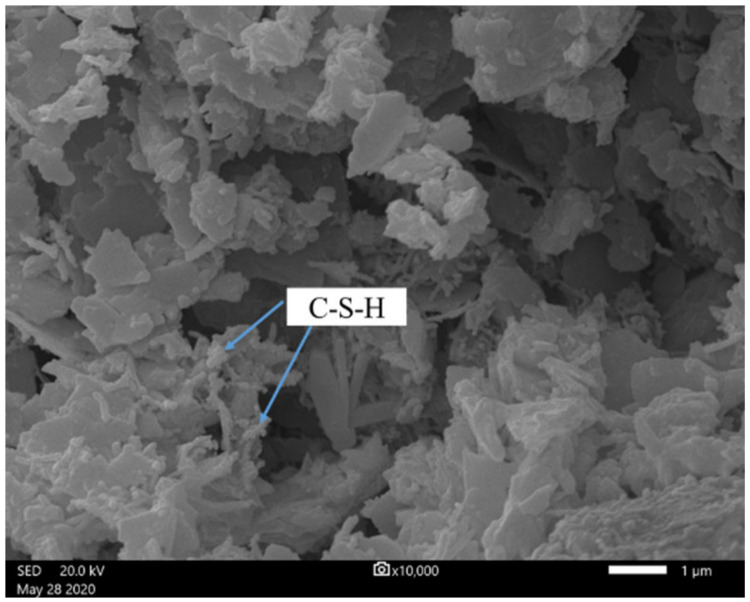
SEM micrograph of CPB with slag.

For CPB prepared with slag and red mud, the specimens had not consolidated after 3 days, and the UCS was less than 0.3 MPa after 7 and 28 days. This is because the hydration degree of slag is extremely low. Slag is a dense byproduct, overall composed of a calcium-rich phase and a silicon-rich phase, and it has a phase separation structure. The weak effect of water molecules is not enough to overcome the decomposition activation energy of the calcium-rich phase, and the calcium-rich phase can maintain its structural stability in water [57]. Only slight hydration reactions can occur on the surface to generate the C-S-H gel. Further hydration reactions are prevented by the low-permeability protective film on the slag surface; thus, water cannot enter the interior of the slag, and the ions inside it cannot move out. Therefore, slag remains almost inert in the water. Although red mud from the Bayer process is strongly alkaline, the bound alkali (Na_2_O and K_2_O) cannot activate its potential hydration property, and thus there are not enough hydration products in the CPB system. As shown in Figure 7, only small amounts of C-S-H can be found in the CPB with slag, which makes the strength low.

#### 3.1.3. Strength Development of Red Mud-Based CPB with Lime

**Figure 8:**
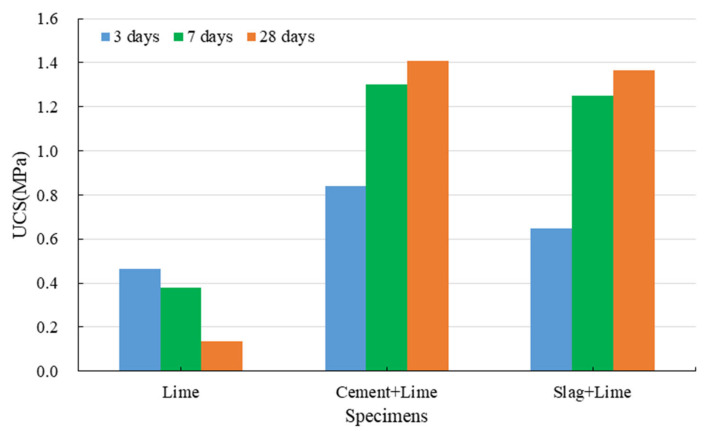
Strength development of red mud-based CPB with lime.

Figure 8 shows that the specimens mixed with lime, and red mud can exhibit a low UCS in 3 days; however, the UCS gradually decreases with the increase in curing time, and even cracks can be clearly observed on the surface of the specimens cured for 28 days. The hydration reactions of lime can consume some water, which accelerates the shrinkage processes of the specimens, and thus these specimens can exhibit the strength in 3 days. In addition to the cohesion between red mud particles, lime plays a vital role in the development of CPB structures. The main effects of lime include its ion exchange, crystallization, and carbonization of calcium hydroxide.

As mentioned previously, the red mud particles with negative charges attract several cations to form an electric double layer, including the low-valent cations, such as Na^+^ and K^+^. The addition of lime generates a large number of calcium ions in the slurry, enhancing the intensity of the counter ions. Due to ion diffusion and the electrostatic attraction, calcium ions would enter the electric double layer and substitute sodium and potassium ions. In addition, the electrostatic repulsion between the counter ions would squeeze the original counter-ions into the adsorption layer. The electrostatic double layer of red mud particles is compressed, which reduces its thickness. In addition, the space between these particles is reduced. The electrostatic repulsion between red mud particles decreases, while the attractive forces between them increase. The interaction between particles transforms from repulsive forces to attractive forces. Therefore, red mud particles can agglomerate.

As the capillary water is gradually consumed and evaporated, the calcium hydroxide in the slurry quickly becomes supersaturated, which promotes the calcium hydroxide to crystallize out of the solutions. The chemical bond can generate crystalline structures at a crystal contact point [58]. The calcium hydroxide crystals overlap and cross other crystals, and the hardening of calcium hydroxide forms a stable protective film on the surface of red mud particles. This improves the interparticle bonding forces and further fills the voids between the particles [59,60,61], so that the overall strength and compactness are improved. When there are a sufficient number of crystals in the hydration products to form a crystalline network structure, the strength of red mud-based CPB increases. Owing to the small amount of calcium hydroxide present in the specimens, the increase in strength caused by the crystallization is not remarkable.

The slurry structures cured in the atmosphere are surrounded by a considerable amount of carbon dioxide. Calcium hydroxide would absorb and react with carbon dioxide to generate calcium carbonate in Equation (2) [62,63,64] and change the physical-mechanical behaviors of the slurry structures. The calcium carbonate crystals may cross each other or coexist with the calcium hydroxide crystals to form a dense crystalline network, which promotes the strength of the hardened structures. In addition, the solid phase volume of calcium carbonate is slightly larger than that of calcium hydroxide, which also makes the hardened structures denser, thereby exhibiting a slight increase in strength. Generally, the carbonization reaction can only be performed under the conditions of appropriate moisture content. In addition, the carbonization starts from the surface of the structures or particles, which is then covered by the formed calcium carbonate, hindering the penetration of carbon dioxide inward and the discharge of internal moisture. Therefore, the carbonization rate of lime is slow, and the effect is mostly manifested on the surface. The main hydration products of the specimens are calcium hydroxide and a small amount of calcium carbonate on the surface. Due to the low strength of calcium hydroxide, the hardened specimens exhibit a low strength.

Ca(OH)_2_ + CO_2_ = CaCO_3_ + H_2_O (2)

However, the specimens become weaker in 28 days, and there are some cracks on the surface. During the specimen hardening, the consumption and evaporation of the capillary water and bound water shorten the distance between solid particles. The specimens show a significant shrinkage deformation, generating internal stress in the specimens. When the internal stress exceeds the tensile strength of CPB, cracks are formed locally, destroying the CPB structures. In addition, the rapid hydration reactions of lime are accompanied by an intense exotherm and significant volume expansion, which destroy the formed crystalline structures during the transition from a slurry to a crystalline phase.

Compared to specimens made of cement, the specimens prepared with cement and lime show a slight decrease in their 3-day and 7-day UCS and a significant reduction in their 28-day UCS. The lime substitutes some cement to reduce the number of hydration products, resulting in a slight decrease in strength at an early stage. Owing to the crystallization pressure and shrinkage of lime, weak planes or cracks are generated inside the specimens. The specimens are first broken along the weak plane or cracks during the UCS tests, and thus the 28-day UCS is significantly reduced.

Regardless of the curing time, the addition of lime significantly increases the UCS of the specimens containing slag. This is mainly because the potential hydration property of slag is activated in the presence of calcium hydroxide [65,66,67]. As an alkaline activator, calcium hydroxide can take the alkaline attack on acidic vitreous in slag at normal temperature, dissociating the slag structures. That is, calcium hydroxide can result in the dispersion and dissolution of slag. Calcium ions on the vitreous surface react with the hydroxide ions in the solutions to form calcium hydroxide, causing the vitreous surface to be destroyed. As the surface structures of the slag are gradually destroyed, hydroxide ions rapidly diffuse into the slag interior and react violently with the active cations to promote the continued collapse of the slag particles. In addition, the active silica and alumina react with the calcium ions in the solutions to form relatively stable hydrated calcium silicate and hydrated calcium aluminate, see Equations (3) and (4). As they are connected to the network structures, the hydration products occupy the space previously filled by water. As the hydration reaction progresses, hydration products are continuously generated in the gaps or capillary pores of the CPB system, and the density of the structures is increased; thus, the strength of red mud-based CPB is continuously increased. Calcium hydroxide not only activates the potential hydration property of slag but also facilitates the hydration products and network structures. However, the potential hydration property of slag has not been completely activated at the early stage, and its hydration rate is low. There are insufficient hydration products in the CPB system, and thus red mud-based CPB shows a low UCS after three days.

3Ca(OH)_2_ + SiO_2_ + 2H_2_O = 3CaO·2SiO_2_·3H_2_O (3)

3Ca(OH)_2_ + Al_2_O_3_ + 5H_2_O = 3CaO·Al_2_O_3_·6H_2_O (4)

### 3.2. Strength Development of Red Mud-Based CPB under the Immersion Conditions

**Figure 9:**
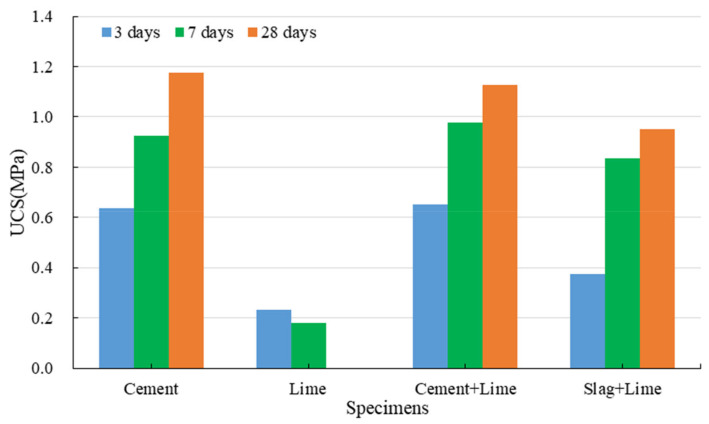
Strength development of specimens under the immersion conditions.

**Figure 10:**
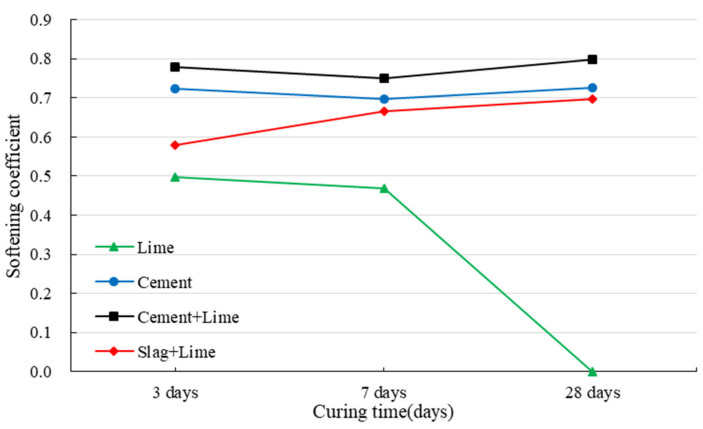
Softening coefficient of red mud based-CPB with different binders.

Figure 9 displays the strength development of red mud-based CPB with different binders under the immersion conditions, and Figure 10 illustrates its softening coefficient with different binders.

**Figure 11:**
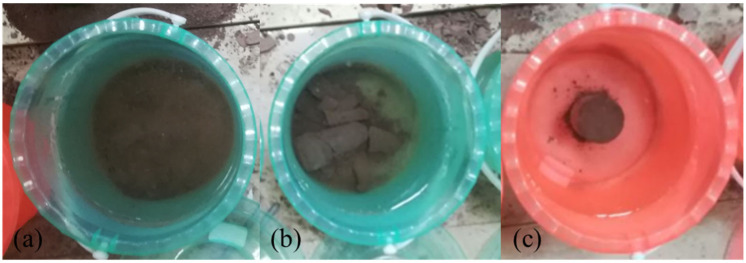
The 28-day specimens immersed in water: (**a**) red mud specimens; (**b**) specimens with lime; (**c**) specimens with cement.

It is well-known that CPB with a higher softening coefficient shows better water stability. As shown in Figure 11a, red mud specimens completely disintegrate after being immersed in water for 30 min, which indicates that red mud from the Bayer process has poor water resistance. In addition, red mud has a fine particle size and contains considerable hydrophilic oxide with a strong ability to absorb water [68,69]. When the consolidated specimens are immersed in water, the mineral compositions are dissolved. In addition, the capillary forces may disappear due to the saturation. The interaction of red mud particles is reduced, and thus the specimens disintegrate and their strength decreases.

The specimens with lime cured for 3 and 7 days show a low UCS after being immersed in water, and the softening coefficient is lower than 0.5. However, the specimens cured for 28 days disintegrate after being immersed in water (Figure 11b). The hydration products of lime are calcium hydroxide, providing weak cementation. In addition, calcium hydroxide is slightly soluble in water. In a dry environment, the crystal contact points remain stable. However, these points generated during the formation of the hardened structures exhibit thermodynamic instability, and due to their deformation and distortion, the crystal lattice at the crystal contact point has a higher solubility than the regular crystal. The crystals in the specimens dissolve in water, causing a significant irreversible reduction in strength. In addition, the cracks provide a channel for water to enter the CPB interior, destroying its structure. Thus, the specimens can be damaged by water.

The specimens with binders can maintain their integrity after being immersed in water (Figure 11c). The specimens with cement have a softening coefficient of 0.71, and those with cement and lime have a softening coefficient of 0.77. All these specimens show better water resistance. Hydration products play a vital role in the development of CPB structures. The pores are gradually filled with hydration products, and the unfilled spaces are gradually divided into capillaries of irregular shapes. The higher the degree of hydration, the denser is the pore structures. This hinders the penetration and diffusion of water into CPB. Calcium hydroxide is the most soluble hydration product and dissolves first. Limited by the amount of water and curing time, only a small amount of hydration products would dissolve in water.

Due to the low degree of hydration, the specimens with slag and lime show a low softening coefficient in 3 days. As the hydration progresses, the softening coefficient is continuously improved.

### 3.3. Leaching Experiments

The specimens cured for 28 days were subjected to leaching experiments, and the results are shown in Table 3. CPB has a significant inhibitory effect on the leaching of hazardous substances in red mud. The leaching concentrations of hexavalent chromium, selenium, fluoride, arsenic, lead, and vanadium are reduced by more than 70%. Compared to the “standards for groundwater quality” (GB/T 14848-2017), all elements except for fluorine can meet the groundwater quality standards.

The inhibitory effect of CPB on hazardous substances is mainly achieved under the solidification and stabilization effects [70]. Under the solidification effect, the binder converts red mud from slurries or particulates into non-flowable and compact blocks with high mechanical strength, solidifying the hazardous substances in CPB through physical wrapping and adsorption [71]. The solidification is primarily a physical effect. As the mechanical strength develops, the hydration products gradually connect to form network structures, and some hazardous substances are encapsulated inside the gel structures. As shown in Figure 5, the hydration products are porous materials that can adsorb molecules of less than the pore size into the capillary pores.

Under the stabilization effect, the binder changes the state or chemical composition of hazardous substances in red mud to reduce its toxicity, solubility, and migration through chemical bonds. In addition, the stabilization is primarily a chemical effect. In addition to being encapsulated in an ionic form, Cr^3+^, Cd^2+^, and Pb^2+^ can also be encapsulated and solidified in the form of solid precipitates [72]. Cr^3+^ and Cd^2+^ can form Cr(OH)_3_ and Cd(OH)_2_ under alkaline conditions, and Pb^2+^ can react with dissolved silicon to form Pb_3_SiO_5_. Other harmful elements, such as As, Sr, Cs, and Mo, are also encapsulated in the solid precipitates.

The heavy metal ions with positive charges can participate in the charge-balancing process. In the CPB system, the aluminum element combines with the four surrounding oxygen atoms in a positive trivalent state, causing the aluminoxy tetrahedron to be negatively charged. Cations, such as Na^+^ or Ca^2+^, are adsorbed around the aluminoxy tetrahedron to balance the charges. Therefore, the cation sites can be substituted by heavy metal ions with a similar radius, thereby stabilizing the heavy metal ions through chemical adsorption.

Ettringite can also produce a stabilizing effect on lead and zinc. In the absence of ${\mathrm{SO}}_{4}^{2-}$, Pb(OH)^3−^ cannot react with Ca^2+^ and AlO^2−^ to form ettringite; however, in the presence of ${\mathrm{SO}}_{4}^{2-}$ in solution, part of Pb^2+^, Zn^2+^, and Cd^2+^ can be captured by ettringite and enter the ettringite crystal lattice. The ettringite exhibits strong chemical adsorption to dissolved Pb^2+^ under alkaline conditions.

The combination of solidification and stabilization effects allows hazardous substances in red mud to form a stable structure, thereby effectively inhibiting the leaching of hazardous substances.

## 4. Conclusions

This study experimentally examined the possibility of utilizing red mud from the Bayer process as the backfill aggregates. The UCS tests, water immersion tests, and leaching experiments were conducted to evaluate the mechanical properties, water resistance, and environmental behaviors of red mud-based CPB with different binders and lime. The following conclusions were drawn:

(1) Red mud from the Bayer process is obviously not an ideal backfilling aggregate due to the fine particle size distribution and strong alkaline. In addition, the specimens made of red mud show poor water resistance and disintegrate after being immersed in water.
(2) Cement can effectively improve the mechanical properties and water resistance of red mud-based CPB. The specimens with cement have a softening coefficient of 0.71, and those with cement and lime have a softening coefficient of 0.77.
(3) Slag cannot be used alone as a binder. However, red mud-based CPB prepared with slag and lime shows excellent mechanical properties and water resistance.
(4) The addition of binders has a significant inhibitory effect on the leaching of hazardous substances in red mud under the solidification and stabilization effects. The leaching concentration of hexavalent chromium, selenium, fluoride, arsenic, lead, and vanadium is reduced by more than 70%.

These findings demonstrate that red mud from the Bayer process can be used for CPB and provide an environmentally friendly and efficient management strategy for red mud in an economical way.

## Figures and Tables

**Table 1:** Physical properties of red mud from the Bayer process.

Specific Gravity	Permeability Coefficient (cm/s)	Median Particle Size (μm)	Specific Surface Area (m^2^/kg)	Nonuniform Coefficient	Curvature Coefficient	pH
2.424	3.35 × 10^−7^	3.248	2940	4.741	0.946	12.1

**Table 2:** Main chemical compositions of cement and slag.

Element Unit	CaO (wt. %)	SiO_2_ (wt. %)	Fe_2_O_3_ (wt. %)	Al_2_O_3_ (wt. %)	MgO (wt. %)	SO_3_ (wt. %)	LOI
Cement	63.20	20.9	2.77	5.45	2.7	2.54	-
Slag	39.25	33.40	0.31	15.15	7.67	2.38	0.11

**Table 3:** Elements’ contents in leaching water (mg/L).

Specimens	Mn	Cu	Zn	Hg	As	Se
Red mud sample	0.002	0.03	0.02	<0.0001	0.007	0.008
Sample with cement	<0.001	<0.01	<0.01	<0.0001	0.003	0.002
Sample with slag	<0.001	<0.01	<0.01	<0.0001	0.002	0.002
Standards	≤0.1	≤1	≤1	≤0.001	≤0.01	≤0.01
Specimens	Cd	Cr6+	Pb	Be	Sb	Ba
Red mud sample	<0.001	0.131	0.01	<0.001	<0.002	<0.01
Sample with cement	<0.001	0.028	<0.001	<0.001	<0.002	<0.01
Sample with slag	<0.001	0.035	<0.001	<0.001	<0.002	<0.01
Standards	≤0.005	≤0.05	≤0.01	≤0.002	≤0.005	≤0.7
Specimens	Ni	Co	Mo	Ag	V	Fluoride
Red mud sample	0.01	0.02	0.11	<0.01	1.05	10.6
Sample with cement	<0.001	<0.001	0.03	<0.01	0.31	2.02
Sample with slag	<0.001	<0.001	0.03	<0.01	0.28	1.75
Standards	≤0.02	≤0.05	≤0.07	≤0.05	-	≤1

## Data Availability

Data are available upon reasonable request.
